# Differential effects of AdOx on gene expression in P19 embryonal carcinoma cells

**DOI:** 10.1186/1471-2202-13-6

**Published:** 2012-01-06

**Authors:** Li Yan, Hai-yong Zhao, Ye Zhang, Yu-fei Shen

**Affiliations:** 1 National Laboratory of Medical Molecular Biology, Department of Biochemistry and Molecular Biology, Institute of Basic Medical Sciences, Chinese Academy of Medical Sciences & Peking Union Medical College, 5 Dongdan Santiao, Beijing 100005, China

## Abstract

**Background:**

Pluripotent cells maintain a unique gene expression pattern and specific chromatin signature. In this study, we explored the effect of the methyltransferase inhibitor adenosine dialdehyde (AdOx) on pluripotency maintenance and gene expression in P19 embryonal carcinoma cells.

**Results:**

After AdOx treatment, the pluripotency-related gene network became disordered, and the early developmental genes were released from the repression. Remarkably, AdOx caused contrasting effects on the expression of two key pluripotency genes, *nanog *and *oct3/4*, with the reduction of the repressive histone marks H3K27me3, H3K9me3 and H3K9me2 only in the *nanog *gene.

**Conclusions:**

Key pluripotency genes were controlled by different mechanisms, including the differential enrichment of repressive histone methylation marks. These data provided novel clues regarding the critical role of histone methylation in the maintenance of pluripotency and the determination of cell fate in P19 pluripotent cells.

## Background

Pluripotent cells, including embryonic stem (ES) cells, embryonic germ cells, and embryonal carcinoma cells, are characterized by their ability to differentiate into all somatic cell types under appropriate conditions [1]. Accordingly, pluripotency-related genes are kept active in pluripotent cells, and early developmental genes are maintained in a 'poised' state (i.e., repressed but ready for activation) [2]. Consistent with the wide array of developmental fates, the chromatin in pluripotent cells is also highly adaptable [3]. The unique gene expression pattern and chromatin signature in pluripotent cells are controlled by a transcription factor network that involves Oct3/4, Sox2, and Nanog [1]. These three core factors are tightly regulated, and even limited fluctuation in their expression may cause significant changes in cell fate [4,5]. In ES cells, the pluripotency-related genes have relatively high levels of the active histone mark H3K4me3 in their chromatin. In contrast, the chromatin that contains poised developmental genes is associated with a combination of H3K4me3 and repressive H3K27me3 marks [2]. Many histone methyltransferases have been proven to be important for normal embryogenesis [6]. Nevertheless, how histone methylation participates in the maintenance of pluripotency and coordinates the precise expression of the core transcription factors remains largely unknown.

P19 pluripotent cell line is derived from mouse embryonal carcinoma that has the ability to contribute to many normal embryonic tissues after blastocyst infection [7-10]. When exposed to nontoxic concentrations of dimethyl sulfoxide (DMSO) in culture conditions, P19 cells can differentiate into muscle cells. When induced with *all trans*-retinoic acid (RA), they can be directed into neuronal lineage cells [11]. AdOx indirectly inhibits S-adenosylmethionine (SAM)-dependent methyl-transfer by inhibiting the hydrolysis of the by-product S-adenosylhomocysteine [12]. AdOx has been broadly used for the functional analysis of protein methylation [13]. It has been shown that AdOx treatment during RA induction interferes with the neuronal differentiation of P19 cells [14].

Here, we show that pre-treatment of AdOx blocks RA-induced P19 cell neuronal differentiation. The impact of AdOx on the expression of pluripotency genes was investigated. We found that in contrast to decreased Oct3/4, the expression of other key pluripotency-related genes is elevated or unchanged in P19 cells treated with AdOx. We then specifically examined the opposing effects of AdOx on the expression of *nanog *and *oct3/4*, which were supported by the differential repressive histone methylation of these genes. These results provide an example on the differential control of pluripotency-related genes by histone methylation in P19 cells.

## Results

### AdOx reduces the neuronal lineage potential of P19 cells

**Figure 1:**
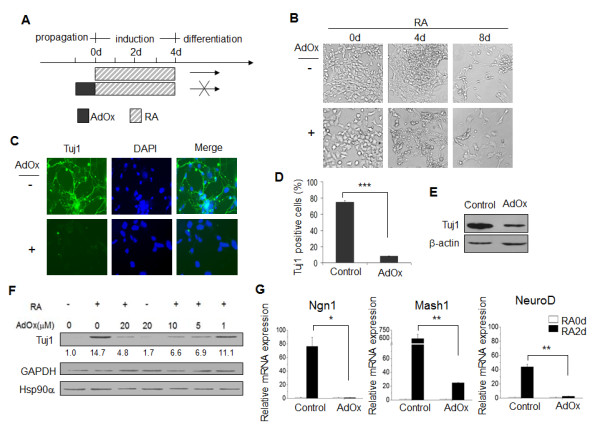
**Impact of AdOx pretreatment on RA-induced differentiation of P19 cells**. **A**. Schematic of the effect of AdOx pre-treatment on RA-induced P19 cells. **B**. Microscopic images showing the impact of AdOx on RA-induced P19 cells. P19 cells were pre-treated with AdOx (+) or without (-) for 1 day (0 d) followed by RA treatment for 4 days (4 d) and then cultured in RA-free medium for another 4 days (8 d). **C**. Immunostaining for Tuj1 (green) in RA-induced P19 cells pre-treated with AdOx (+) or without (-). **D**. Quantification of Tuj1-positive P19 cells induced by RA and pretreated with AdOx (AdOx) or without (control). **E**. Impact of AdOx pretreatment on the expression of Tuj1 and β-actin in RA-induced P19 cells as shown in Western blots and described as in D. **F**. Impact of AdOx concentration on the expression of Tuj1 in P19 cells induced with RA (+) or without (-) as shown in Western blots. GAPDH and Hsp90α are the loading controls. Numbers indicated quantification of the Tuj1 western signal relative to GAPDH. Digits at the bottom of the first row indicates the relative darkness of each band compared with the control lane set as 1.0. **G**. RT-qPCR analyses of *ngn1, mash1*, and *neuroD *mRNA expression in P19 cells pre-treated with AdOx (AdOx) or without (Control) for 1 day (RA 0 d) and then induced with RA (RA 2 d). Each bar in the histograms of this figure represents a mean value with S.D. from three independent experiments (mean ± SD). Asterisks represent significance in statistics: (*) as P < 0.05, (**) for P < 0.01 and (***) for P < 0.001.

To explore whether AdOx affects the maintenance of pluripotency, we examined the neuronal differentiation potential of AdOx-treated P19 cells. P19 cells were pre-treated with AdOx for 1 day and then induced by RA as shown in Figure 1A. Our data showed that AdOx pre-treated cells neither aggregated by day 4 nor formed neuron-like networks by day 8, in contrast to AdOx-null RA-treated control cells (Figure 1B, bottom *vs*. top panels). Furthermore, immunostaining showed that AdOx significantly reduced the expression of neuron-specific tubulin-isotype β-tubulin III (Tuj1) in RA-induced P19 cells (Figure 1C, bottom *vs*. top panels). In contrast to the almost three quarters of RA-induced cells that express Tuj1 protein and have extended neurites, only 8% of AdOx-treated cells were Tuj1-positive, and they had fewer neurites (Figure 1D). Similar results demonstrating the inhibitory effects of AdOx on RA-induced expression of Tuj1 were obtained by Western blotting (Figure 1E). The reduction of Tuj1 was dependent on the dosage of AdOx (Figure 1F). Next, we investigated whether the neuronal differentiation program can be initiated by AdOx pre-treatment. The hierarchical expression of the transcription factors Ngn1, Mash1, and NeuroD is essential for neuronal fate determination [15]. We found that the RA-induced expression of these early neuronal transcription factors was remarkably reduced or completely abolished at the mRNA level in the presence of AdOx (Figure 1G). These results indicated that the potential for neuronal fate is severely reduced in AdOx-pretreated P19 cells.

### The impact of AdOx on the expression of pluripotency-related genes in P19 cells

**Figure 2:**
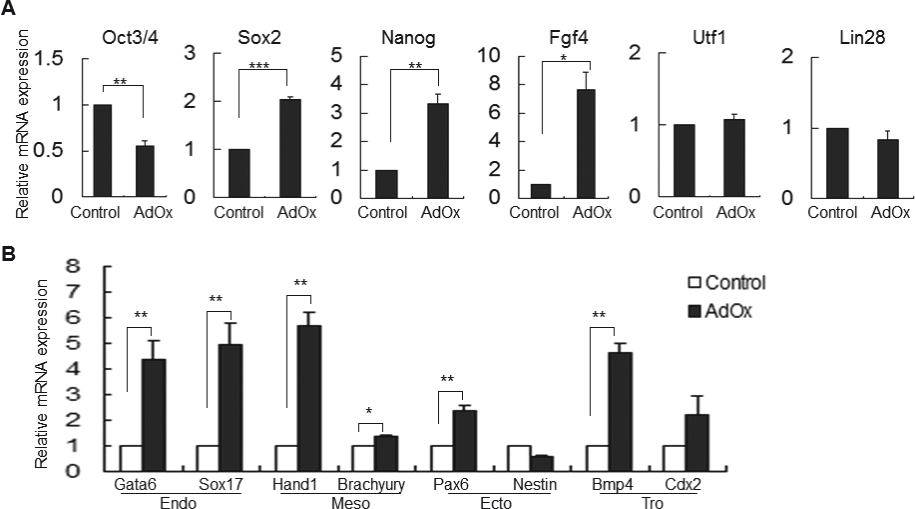
**Impact of AdOx on the expression of pluripotency-related and germ layer marker genes in P19 cells**. **A**. RT-qPCR analysis of pluripotency-related genes in P19 cells pretreated with AdOx for 1 day. The mRNA levels corresponding to each gene are indicated on top of each panel. **B**. RT-qPCR analysis of the mRNA levels of the endodermal (Endo), mesodermal (Meso), ectodermal (Ecto) and trophectodermal (Tro) marker genes. Annotations for Figure 2A & 2B are as described for Figure 1G.

It is important to determine whether the effect of AdOx is correlated with the maintenance of pluripotency. We thus examined the expression of pluripotency-related factors, including Oct3/4, Sox2, Nanog, Fgf4, Utf1, and Lin28. The analysis showed that Oct3/4 mRNA was reduced, which is consistent with the reduced differentiation potential. Unexpectedly, Sox2, Nanog, and Fgf4 were markedly up-regulated after AdOx treatment (Figure 2A). As a control for the pluripotency-related genes, we examined the impact of AdOx on the mRNA expression of three germ layer-marker genes as well (Figure 2B), because these genes are maintained in a 'poised' state in pluripotent cells that are expressed only when the three layers are established during early embryonic development [2]. The mRNA levels of almost all of these genes in P19 cells increased with AdOx treatment, whereas the mRNA level of *nestin*, an ectoderm marker, did not obviously change. Collectively, these data show that the expression pattern of pluripotency-related genes is disordered in AdOx-treated P19 cells and that early development-related genes are released from their poised states

### AdOx has opposite effects on the expression of Nanog and Oct3/4

**Figure 3:**
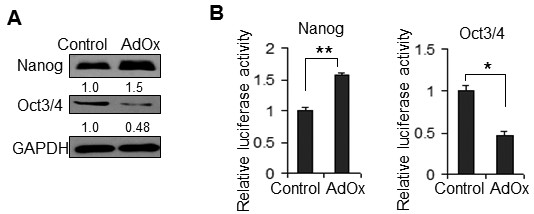
**Impact of AdOx on the expression of Nanog and Oct3/4 in P19 cells**. **A**. Nanog and Oct3/4 expression in P19 cells with AdOx (AdOx) or without (Control) as determined by Western blot. GAPDH is a loading control. Numbers indicated quantification of the Nanog or Oct3/4 western signal relative to GAPDH respectively. Digits at the bottom of the first & second row indicate the relative darkness of each band compared with the control lane set as 1.0. **B**. Impact of AdOx on the relative luciferase activity of the *nanog *and *oct4 *promoters. pGL3-*nanog*-luc (driven by the -289/+117 fragment of the *nanog *gene) and pGL-*oct3/4*-luc (driven by the -2143/+30 fragment of the *oct4 *gene) were individually co-transfected with pRL-TK into P19 cells treated with AdOx or without. The relative promoter activities showed that AdOx has a different effect on the two genes in P19 cells. Annotations are as described in Figure 1G.

Next, we focused on the differential regulation of pluripotency-related genes by AdOx. We examined the protein expression of the core transcription factors Oct3/4 and Nanog. AdOx-induced changes in the protein levels of Nanog and Oct3/4 were similar to those observed at the mRNA level (Figure 3A*vs*. 2A). Reporter plasmids driven by the *nanog *(-289/+117) and *oct3/4 *(-2143/+30) promoters were then used to confirm the effects of AdOx. It has been reported that these two reporter plasmids can recapitulate the endogenous expression of *nanog *and *oct3/4 *[16,17]. We found that the reporter activity driven by the *nanog *promoter was increased, whereas that of *oct3/4 *was reduced in AdOx-treated P19 cells (Figure 3B). Because they are consistent with the measured mRNA levels, the promoter activity data suggest that AdOx has opposite effects on the transcription of the *nanog *and *oct3/4 *genes.

### Impact of AdOx on the occupation of histone H3K27me3 and H3K9me3 on the nanog and oct3/4 genes

**Figure 4:**
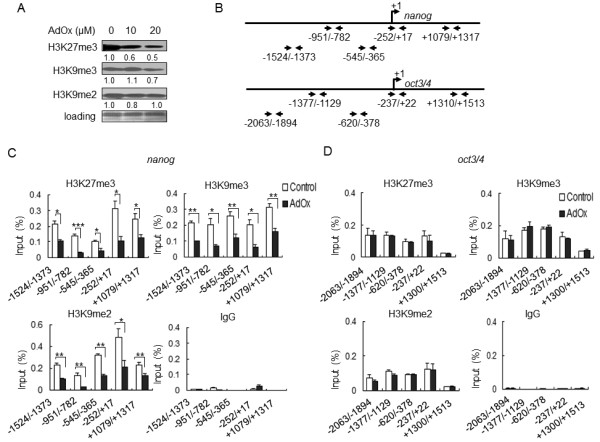
**Impact of AdOx on H3K27me3, H3K9me3 & H3K9me2 marks on the nanog and oct3/4 genes in P19 cells**. **A**. P19 cells were treated with the indicated concentrations of AdOx, and the nuclear extracts were analyzed by Western blot with the indicated antibodies for specific histone methylation marks. Numbers indicated quantification of the H3K27me3, K9me3, or K9me2 western signal relative to loading respectively. Digits at the bottom of the top three rows indicates the relative darkness of each band compared with the control lane set as 1.0. **B**. Positions of the primer pairs used in ChIP assays for analyzing the repressive methylation marks on the *nanog *and *oct3/4 *genes. **C & D**. Effect of AdOx on H3K27me3, H3K9me3, or H3K9me2 levels on the *nanog *(C) and *oct3/4 *(D) genes in P19 cells. The positions of the methylated histone marks to the genes are indicated at the bottom of each group shown in ChIP assays. IgG is a negative antibody control. Annotations are as described in Figure 1G.

Methylation of histone H3, including H3K27me3, H3K9me3, and H3K9me2, is usually associated with the gene repression. We next examined the global effects of AdOx on these repressive methylation marks and found that AdOx reduced the level of H3K27me3 and H3K9me3, but not H3K9me2, in P19 cells (Figure 4A). To examine whether the *nanog *and *oct3/4 *promoters are associated with repressive methylation marks, ChIP assays were carried out for H3K27me3, H3K9me3, and H3K9me2 on five genomic sites that were indicated on the *nanog *and *oct3/4 *genes (Figure 4B). The ChIP data revealed that all three repressive marks were significantly reduced around the *nanog *gene after AdOx treatment (Figure 4C), but none were affected in the *oct3/4 *gene (Figure 4D). This result suggested that the AdOx-mediated reduction in repressive methylation marks on the *nanog *gene (but not the *oct3/4 *gene) is correlated with the differential regulation of these two major pluripotency-related genes by AdOx in P19 cells.

## Discussion

In this study, we demonstrate that AdOx affects the maintenance of pluripotency in P19 cells. Among the pluripotency-related genes, nanog and oct3/4 are differentially regulated by AdOx, and their regulation is correlated with a differential reduction in repressive histone methylation marks in these genes.

The SAM-dependent methyltransferase substrates include lipids, nucleic acids, and a variety of proteins [13]. The effects of AdOx may be universal. After treatment of P19 cells with AdOx, we observed that in addition to the aberrant pluripotent gene expression pattern, AdOx blocked the cell cycle, changed the cell shape and induced expression of the adhesive molecule E-cadherin (data not shown). All of these effects may have contributed to the loss of neuronal potential. In addition, the nonspecific release of early developmental genes may also have disturbed the potential for neuronal fate in P19 cells.

In this study, we examined the expression of six pluripotency-associated genes in P19 cells and their changes in response to AdOx treatment. Utf1 and Lin28 were not responsive to AdOx. In contrast, Sox2, Nanog, and Fgf4 showed higher expression levels following AdOx treatment, whereas the expression of Oct3/4 was reduced. Nanog, Oct3/4 and Sox2 are all critical transcription factors for pluripotency maintenance, and Sox2 functions to maintain the identity of neuronal progenitors, suggesting that the core pluripotency transcription factors perform distinct roles during embryonic development [18]. Recently, it was reported that Sox2 and Oct3/4 have opposite effects in defining mesendoderm and neural ectoderm [19]. We found that Nanog and Oct3/4 were differentially regulated by AdOx, which suggests that they are controlled by different mechanisms. Consistent with this finding, Nanog and Oct3/4 play non-overlapping roles in the establishment of the primitive endoderm and the epiblast [20]. These clues led us to study the histone methylation of these two genes.

To maintain pluripotency, the crucial pluripotency transcription factors are maintained at relatively high but precisely controlled levels [4,5]. In ES cells, the localized chromatin regions of poised developmental genes have both the active histone mark H3K4me3 and the repressive H3K27me3 mark, whereas the pluripotency-related genes are mainly occupied by relatively high levels of H3K4me3 [2]. However, we found repressive histone methylation marks at pluripotency-related genes at low levels, implying that they potentially have a role in the modulation of pluripotency-related genes. The expression of Nanog in ES cells requires the histone demethylase Jmjd2C to antagonize the repressive H3K9me2 [21]. Our ChIP data revealed that AdOx treatment preferentially causes decreased levels of the three repressive histone methylation marks H3K9me2, H3K9me3, and H3K27me3 on the 5' proximal region of the *nanog *gene, whereas it has no effects on the *oct3/4 *gene. In other words, the repressive histone methylations are more involved in the regulation of *nanog *than in the regulation of *oct3/4 *in P19 cells. As reported previously, both *nanog *and *oct3/4 *are under the control of very similar composite sox-oct elements consisting of neighboring binding sites for Sox2 and Oct3/4 [16,22]. These observations raised interesting questions: how is the differential regulation of *nanog *and *oct3/4 *achieved? Is the underlying mechanism related to functional differences between Nanog and Oct3/4? We expect that further investigations in the fields of ES cells and developing embryos would confirm our results and answer the above questions. Because AdOx may also influence the active histone methylation marks, the methylation of DNA and on non-histone proteins [13], more specific manipulations on the relevant histone methyltransferases would indeed helpful in elucidating the differential regulation of *nanog *and *oct3/4*.

## Conclusions

We have shown that the expression of Nanog and Oct3/4 are differentially regulated by AdOx, with the repressive histone marks reduced only on the *nanog *gene. These data shed light on the critical role of histone methylation in the maintenance of pluripotency and the determination of cell fate in pluripotent cells.

## Methods

### Cell culture and treatment

P19 cells were cultured in minimum essential medium (MEM) (Invitrogen) supplemented with 10% (v/v) fetal calf serum. For neuronal differentiation, P19 cells were cultured in medium containing 0.5 μM RA (all trans-retinoic acid, Sigma) for 4 days, and the aggregates were plated as a monolayer and cultured for another 4 days in the absence of RA [23]. AdOx pre-treatment involved adding 20 μM of AdOx (Sigma) to P19 cells for 1 day. Then, AdOx was removed, and the cells were cultured with RA.

### Antibodies

The following antibodies were used: monoclonal antibody against Neuronal Class III β-tubulin (Tuj1) from Covance, Inc. (MMS435P); antibody against β-actin (ab8226) from Abcam; antibody against HSP90α from Stressgen (SPS-771); antibodies against GAPDH (MAB347) and Sox2 (AB5603) from Chemicon; antibody against Nanog from BETHYL (A300-397A); antibody against Oct3/4 from Santa Cruz (sc-5279); antibodies against trimethyl-Histone H3 (Lys27, 07-449), trimethyl-Histone H3 (Lys9, 07-442), dimethyl-Histone H3 (Lys9, 07-212), and IgG (12-370) from Upstate Biotech.

### Quantitative Real Time RT-PCR analysis

Quantitative Real time RT-PCR assays were carried out as previously described [24]. The relative expression of genes was normalized against GAPDH, using the comparative CT method according to the manufacturer's instructions (Roter-Gene RG-3000A Real-time PCR System, Corbett Research, Australia). Primers used in the PCR assays are shown in Table 1. The experiments were repeated at least three times, with statistical analyses for each individual experimental set. All the values in the experiments were expressed as the mean ± S.D.

**Table 1:** Primers used in q-RT-PCR analysis

Gene	Forward primer	Reverse primer
*ngn1*	CCCTGAAGACGAGGTGAAAAGT	CTTGCCATTGTATTGTCAGCCG

*mash1*	TAACTCCCAACCACTAACAGGC	TGAGGAAAGACATCAACCCAGT

*neuroD*	TTCCACGTCAAGCCGC	TCGGCGGATGGTTCGT

*sox2*	CCACCAATCCCATCCA	ACCGCCTAACGTACCACT

*oct3/4*	TCACTCACATCGCCAATCA	GTAGCCTCATACTCTTCTCGTT

*nanog*	GGCAGCCCTGATTCTTCTAC	CGCTTGCACTTCATCCTTT

*lin28*	CGGCCAAAAGGGAAGAACAT	CATTCCTTGGCATGATGGTCT

*fgf4*	GGCTTCGGCGGCTCTACT	AGGATTCGTAGGCGTTGTAGTTG

*utf1*	TCCTCTTACGAGCACCGACAC	GCAACGCGGTATTCAACGA

*gata6*	AACAGCAGTGGCTCTGTCCC	CTCGGGGTTGGCGTTTT

*sox17*	TGGGCCAAAGACGAACG	TTGGGGTGGTCCTGCATAT

*hand1*	CTACTTGATGGACGTGCTGG	CAACTCCCTTTTCCGCTTG

*brachyury*	CCAGTCTCTGGTCTGTGAGCA	GGGGAGCCTCGAAAGAA

*pax6*	GTGAATGGGCGGAGTTATG	ACTTGGACGGGAACTGACA

*nestin*	TGAGAACTCTCGCTTGCAGAC	GTATCCCAAGGAAATGCAGC

*bmp4*	CGAGCCAACACTGTGAGGA	GAGGTTGAAGAGGAAACGAAA

*cdx2*	TCAGGAGGAAAAGTGAGCTG	GGCTGTGGAGGCTGTTGTT

*gapdh*	GAAGGTGAAGGTCGGAGTC	GAAGATGGTGATGGGATTT

### Western blotting

Whole cell extracts (WCEs) were prepared as described previously [23]. The pellets were washed twice with RIPA buffer and then resuspended in 500 μl RIPA buffer (containing protease inhibitors). After adding SDS to 1%, samples were sonicated for 5 seconds on ice on high; the clear solutions were then stored as "nuclear extracts". The samples were separated in SDS-polyacrylamide gels and analyzed as described previously [25]. The density of bands was analyzed and normalized with internal controls (GAPDH or loading) using AlphaImager™ 2200 (Alpha Innotech Corp).

### Immunofluorescence

The cells were first fixed with 2% paraformaldehyde, permeabilized with 0.2% Triton X-100 in PBS for 10 min and then washed and blocked in 1% goat serum. The cells were consecutively incubated in 1:100 diluted mouse anti-Tuj1 antibody at 4°C overnight and the fluorescein isothiocyanate (FITC)-conjugated secondary antibody (1:100) at 37°C for 30 min. The cells were then washed and incubated in DAPI (1:3000) for 10 min followed by two PBS washings. Images were collected using a Nikon Eclipse TE2000-U microscope. DAPI was used to stain the nuclei to count the number of cells. The percentage of cells that were immunoreactive for Tuj1 antigens was determined by capturing images of random fields. DAPI-stained nuclei and cells positive for Tuj1 protein were counted.

### Luciferase reporter assays

The pGL3-*nanog*-luc (-289/+117) was from Dr. Paul Robson [16]. The pGL-*oct3/4*-luc (-2143/+30) was from Dr. Duanqing Pei [17]. P19 cells were transfected with VigoFect Reagent (Vigorous Biotech) according to the manufacturer's instructions. pGL3-*nanog*-luc or pGL-*oct3/4*-luc reporter plasmids (0.1 μg) and 0.002 μg of control plasmid pRL-TK were co-transfected into the cells. Twenty-four hours after transfection, the medium was replaced with fresh α-MEM (AdOx- or AdOx+). Promoter activity was measured 24 hours later. The activities of both luciferase reporters were determined by the Dual-Luciferase Reporter System (Promega) according to the manufacturer's instructions. The relative luciferase activities, as measured in relative light units, were compared to a co-transfected internal control (pRL-TK). The assay results are shown as the means ± S.D.

### Chromatin immunoprecipitation

Chromatin immunoprecipitation (ChIP) assays were carried out as described earlier [24]. The primers used in ChIP-qPCR assays are shown in Table 2. The cycle quantity required to reach a threshold in the linear range (Qt) was determined and compared with a standard curve for the primer set generated by five 10-fold dilutions of genomic DNA samples of known concentration. The percentage of immunoprecipitated DNA relative to input (Input %) was calculated and shown as the mean ± SD from three independent experiments.

**Table 2:** Primers used in ChIP-qPCR analysis

Gene	Forward primer	Reverse primer
*nanog *-1524/-1373	AGAGGCAGACAGTGGTGACGA	CCACAGCCACCATACTACTACTG

*nanog *-951/-782	GGCAAACTTTGAACTTGGGATGTGGAAATA	CTCAGCCGTCTAAGCAATGGAAGAAGAAAT

*nanog *-545/-365	GCTCTTTCTTCAGACTTGCGTT	GAGTTTCCTCTTCCATTCTTTCTAC

*nanog *-252/+17	GGGGATGTCTTTAGATCAGAGG	CCAAATCAGCCTATCTGAAGG

*nanog *+1079/+1317	TGTCCCCTTTTTTCTGAGCTT	CACTTTTCCCACCTCCAAAAT

*oct3/4 *-2063/-1894	GGAACTGGGTGTGGGGAGGTTGTA	AGCAGATTAAGGAAGGGCTAGGACGAGAG

*oct3/4 *-1377/-1129	TGCTCTGGGCTTTTTGAGGCTGTGTGATT	TGGCGGAAAGACACTAAGGAGACGGGATT

*oct3/4 *-620/-378	TCTACCAACCTGGACAACACAAGATGGAA	GCCACTCCTCAGTTCTTGCTTACCCAC

*oct3/4 *-237/+22	GTGAGGTGTCCGGTGACCCAAGGCAG	CGGCTCACCTAGGGACGGTTTCACC

*oct3/4 *+1310/+1513	GGAGTCCCCTAGGAAGGCATTAATAGTTT	GGATTCTCTCGGCTTCAGACAGACTTT

### Statistical analysis

Statistical analyses were performed using a two-tailed Student's *t*-test. All of the data are shown as means with standard deviations. P < 0.05 was considered significant (*), p < 0.01 was considered highly significant (**) and P < 0.001 was considered extremely significant (***).

## Authors' contributions

LY, H-YZ, YZ, Y-FS conceived the study, designed the experiments; LY and H-YZ performed the experiments and data analysis; LY, H-YZ, YZ, Y-FS read and revised the final manuscript.
